# Supplementary material for: Synthesis and Characterization of Healable Waterborne Polyurethanes with Cystamine Chain Extenders
Source: Molecules. 2019 Apr 16;24(8):1492. doi: 10.3390/molecules24081492 (PMC6515350; doi:10.3390/molecules24081492)
Supplement: Supplementary file 1 [file molecules-24-01492-s001.pdf]

SUPPORTING INFORMATION

Synthesis and Characterization of Healable Waterborne Polyurethane with Cystamine Chain Extenders

Dae-Il Lee†, Seung-Hyun Kim†, and Dai-Soo Lee*

Division of Semiconductor and Chemical Engineering, Chonbuk National University, Baekjedaero 567, Deokjin-gu, Jeonju, Chonbuk, 54896, Korea; ldi4084@naver.com(DIL); thdnlsla@naver.com(SHK)

† Contributed equally to this work

* Correspondence: daisoolee@jbnu.ac.kr; Tel.: +82-63-270-2310

Table S1. Tensile strength and healing efficiency of WPUS reported in literatures.

Functional groups for self-healing WPU	Tensile strength (MPa)	Healing efficiency (%)	Reference
Disulfide (aromatic)	18	Not available	[32]
〃	4.5	90.7	[29]
〃	23.1	Not available	[31]
Disulfide (aliphatic)	0.74	90.5	[28]
Diels-Alder reaction adduct	9.3	92.5	[5]
Host-Guest of cyclodextrin/polyethylene glycol	22.5	91	[10]
Exchange reaction (amine/urea)	3	Not available	[11]
Cycloaddition of coumarin	2	78.7	[9]
Graphene (GO)	27.1	85	[6]
Ionic group	8.57	Not available	[8]

Table S2. Average particle size and molecular weights* of WPU dispersion.

Sample code	Number average diameter (nm)	Molecular weight (M_n)	Molecular weight (M_w)
WPU-EDA	40.8	17,861	42,308
WPU-cystamine	524.0	20,822	45,705

* Average molecular weights of the samples dissolved in DMF/THF(1:1 by wt) were measured by gel permeation chromatography (GPC, Agilent 1200S, Agilenst Technologies, Palo Alto, USA).

Table S3. Mechanical properties and self-healing efficiencies after cut-and-healing test of WPU-cystamine films.

Healing temperature (°C)	Mechanical Properties and Healing efficiencies	Healing time (hr)			
		1	3	6	12
110	Stress (MPa)	4.9	5.4	5.4	4.0
	Strain (%)	12	12	14	11
	Healing efficiency (%)	26	28	28	21
130	Stress (MPa)	5.4	7.6	5.7	5.6
	Strain (%)	18	36	18	15
	Healing efficiency (%)	28	40	30	29

Table S4. Glass transition temperature of heat-treated WPU-EDA.

Heat treatment time (hr)	Heat treatment temperature (°C)			
	110		130	
	T_{gs}	T_{gh}	T_{gs}	T_{gh}
0	-78.6	54.5	-78.6	54.5
1	-79.5	52.4	-79.5	51.2
3	-79.0	51.2	-80.3	47.2
6	-80.1	47.9	-80.4	46.3
12	-79.5	47.3	-79.6	46.2

Table S5. Glass transition temperature of heat-treated WPU-cystamine.

Heat treatment time (hr)	Heat treatment temperature 110 (°C)		Heat treatment temperature 130 (°C)	
	T_{gs}	T_{gh}	T_{gs}	T_{gh}
0	-78.6	54.5	-78.6	54.5
1	-79.1	52.1	-79.1	51.3
3	-78.8	51.2	-79.9	49.8
6	-79.6	47.5	-79.0	46.9
12	-79.0	46.0	-79.2	45.9

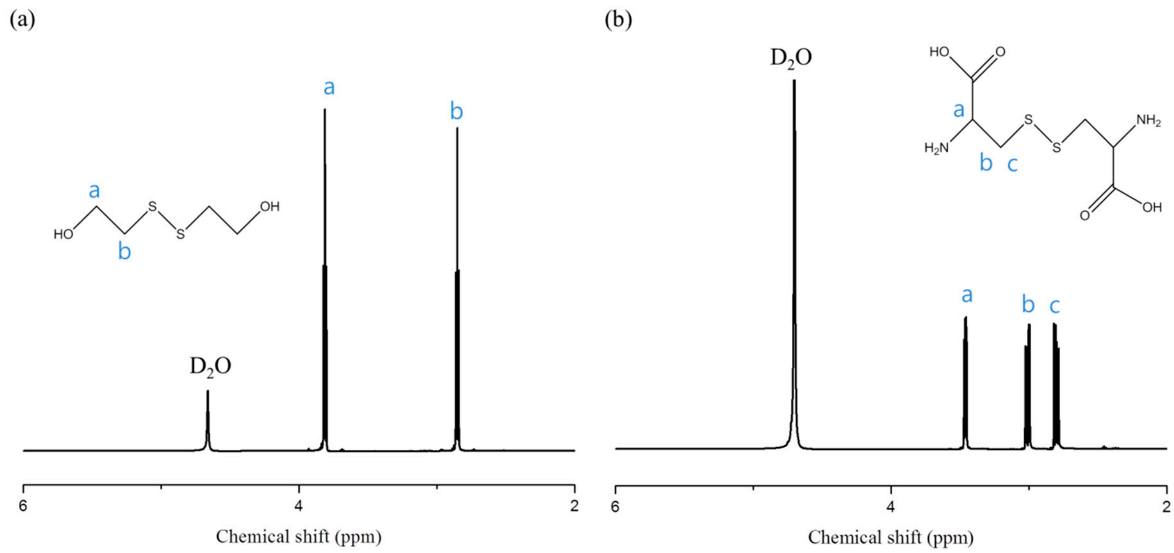

Figure S 1. ^{1}H NMR spectra of the model compounds in the disulfide metathesis reaction: (a) Disulfide 1; (b) Disulfide 2.

Disulfide 1: ^{1}H NMR(600 MHz, D_2O, δ/ppm): 2.85(t,4H,-CH_2-), 3.81(t,4H,-CH_2-).
Disulfide 2: ^{1}H NMR(600 MHz, D_2O, δ/ppm): 2.80(m,2H ,-CH_2-), 3.01(m,2H,-CH_2-), 3.46(m,2H ,-CH-).

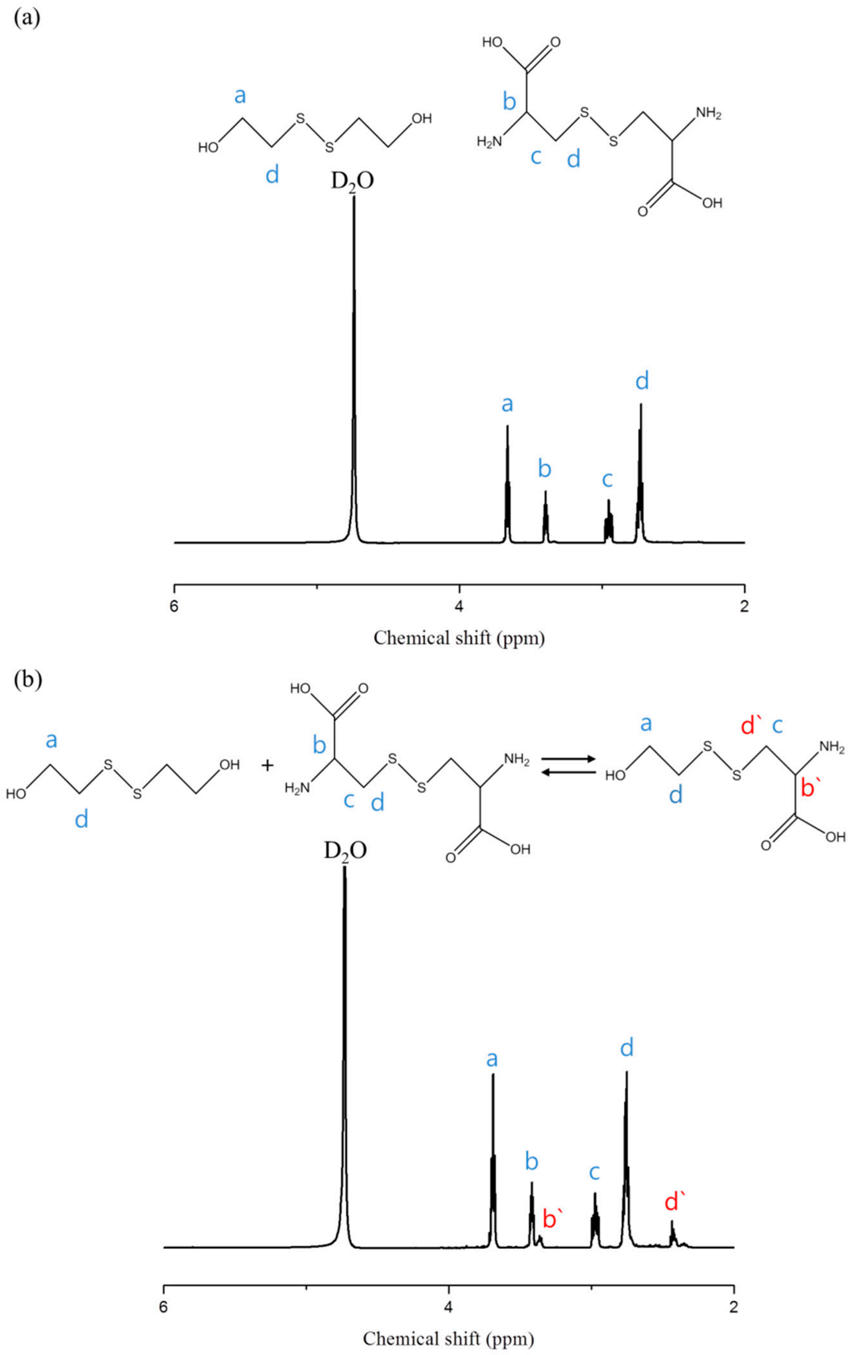

Figure S 2. ^1H NMR spectra of the model compounds in the disulfide metathesis reaction: (a) mixture of Disulfide 1 and Disulfide 2; (b) Product.

A mixture of Disulfide 1 and Disulfide 2: ^1H NMR(600 MHz, D_2O, δ/ppm): 2.62(m,6H,-CH_2-), 2.84(m,2H,-CH_2-), 3.28(m,2H ,-CH-), 3.56(m,4H ,-CH_2-).
Product: ^1H NMR(600 MHz, D_2O, δ/ppm): 2.32(m,0.3H,-CH_2-), 2.62(m,5.7H,-CH_2-), 2.84(m,2H,-CH_2-), 3.24(m,0.3H,-CH_2-), 3.28(m,1.7H ,-CH-), 3.56(m,4H ,-CH_2-).

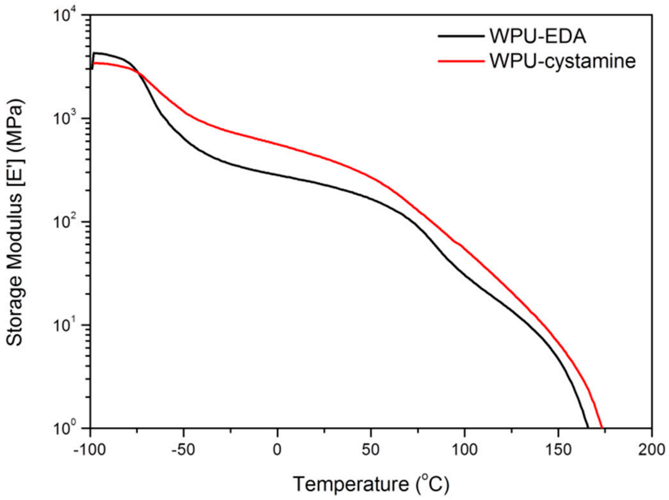

Figure S 3. Storage moduli of the WPU films.

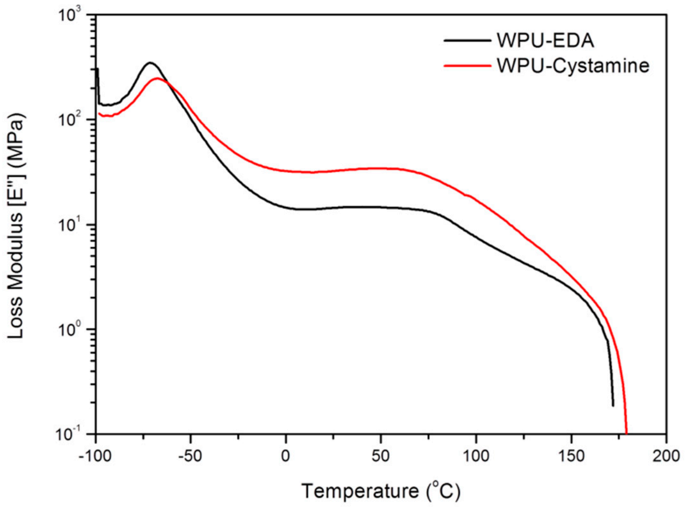

Figure S 4. Loss moduli of the WPU films.

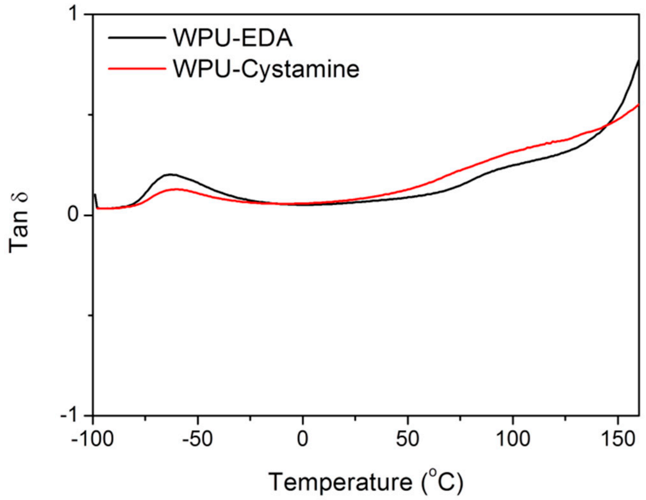

Figure S 5. Tan δ of the WPU films.

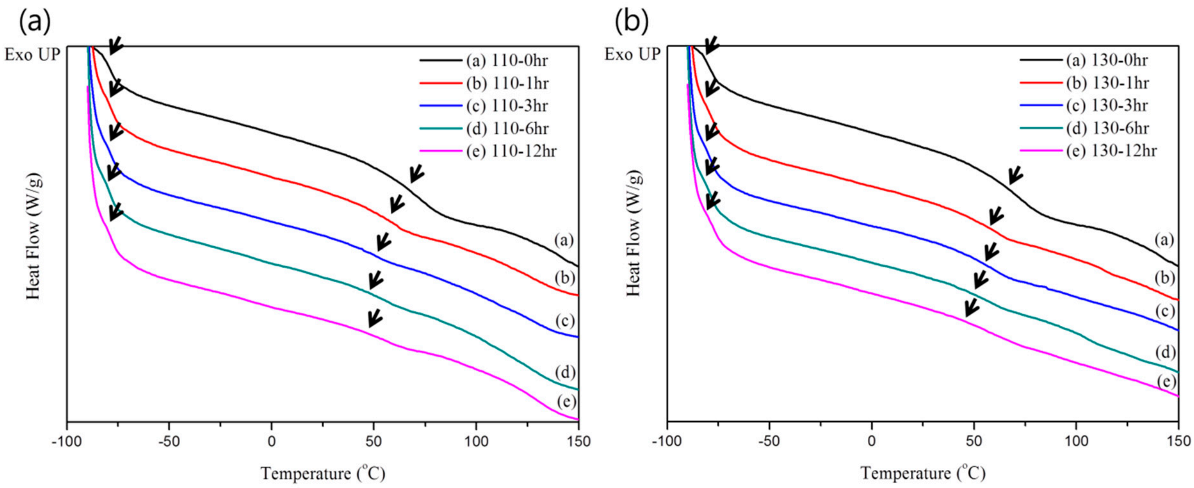

Figure S 6. Thermal properties of WPU-EDA during heat treatment at different temperatures (°C): (a) 110; (b) 130.

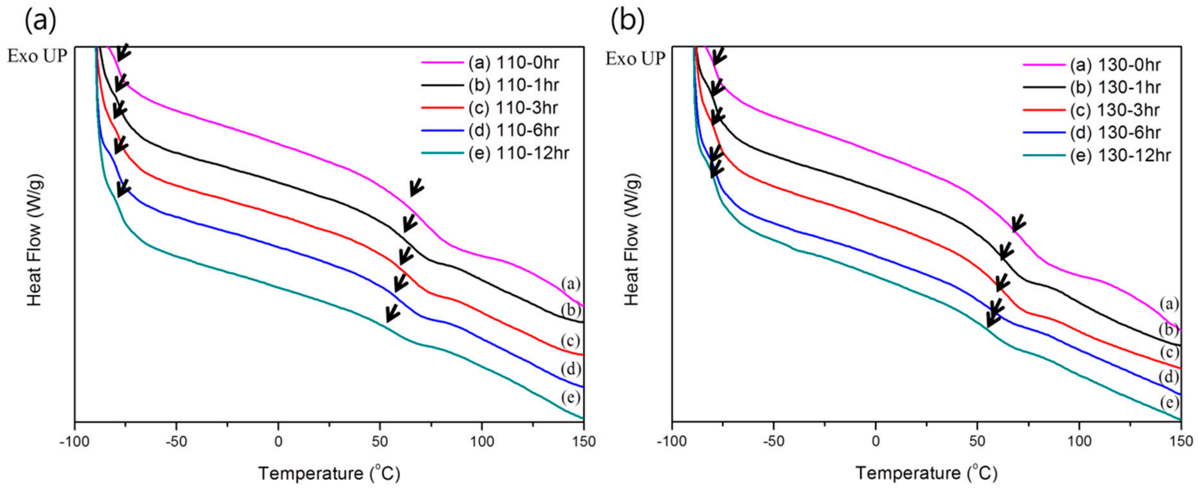

Figure S 7. Thermal properties of WPU-cystamine during heat treatment at different temperatures (°C): (a) 110; (b) 130.

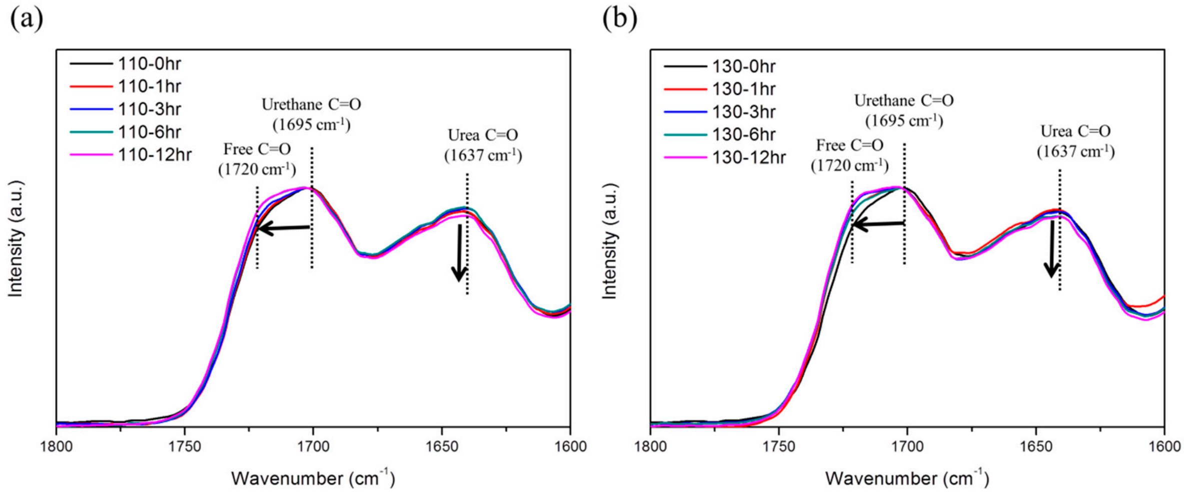

Figure S 8. FT-IR spectra of WPU-EDA during heat treatment at different temperatures (°C): (a) 110; (b) 130.

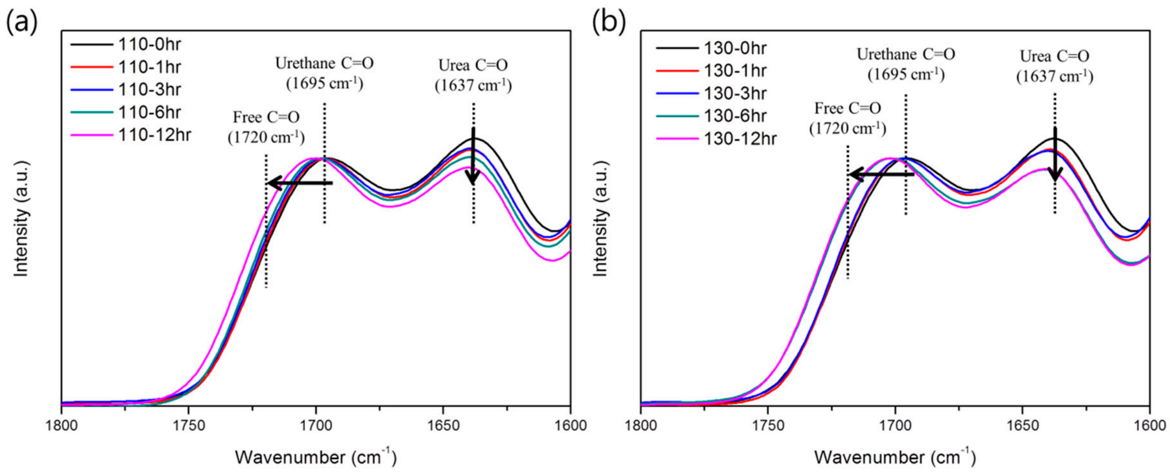

Figure S 9. FT-IR spectra of WPU-cystamine during heat treatment at different temperatures (°C): (a) 110; (b) 130.

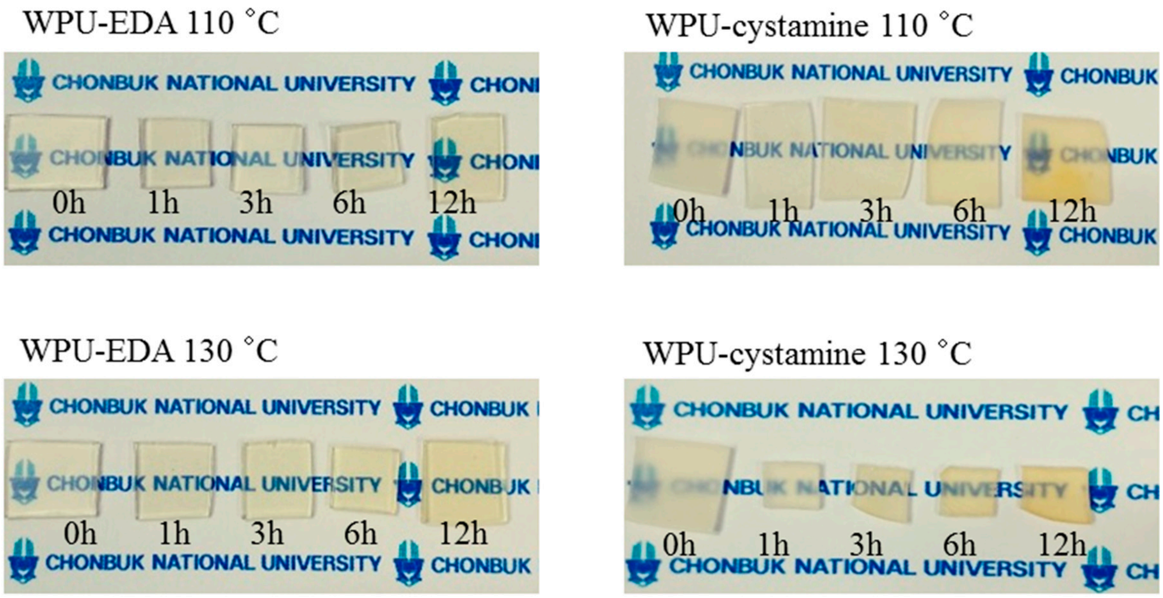

Figure S 10. Photographs of WPU films during the heat treatments.

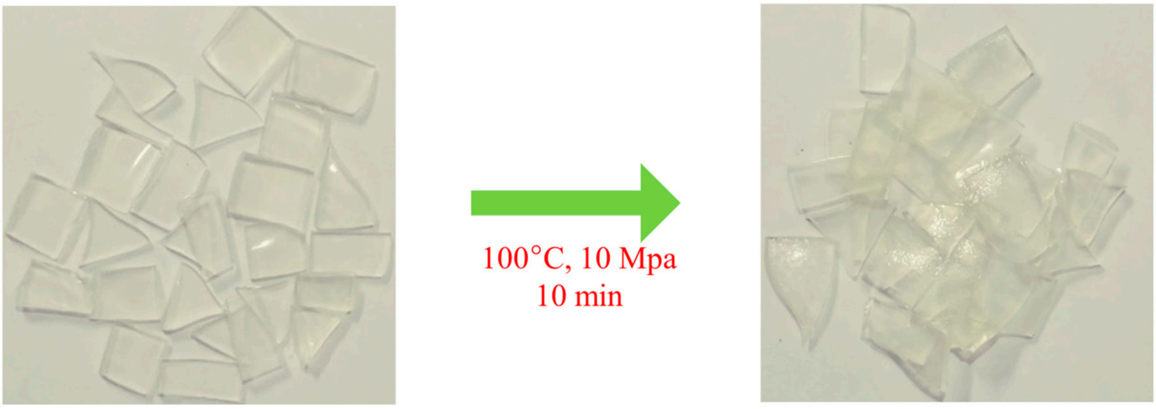

Figure S 11. Reprocessing of the WPU-EDA films.

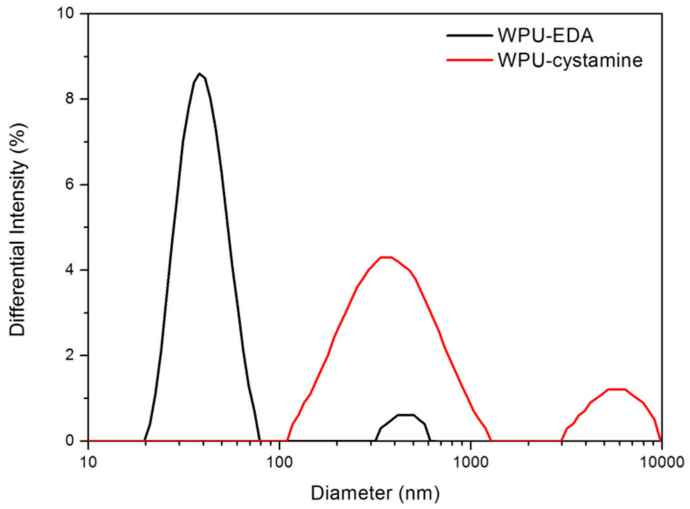

Figure S 12. Particle size distribution of WPU dispersion determined by Particle Size Analyzer (UPA-150, Microtrac, Montgomeryville, USA) .
